# Learning progression toward a measurement concept of fractions

**DOI:** 10.1186/s40594-018-0119-2

**Published:** 2018-06-27

**Authors:** Jesse L. M. Wilkins, Anderson Norton

**Affiliations:** 1 School of Education, Virginia Tech, War Memorial Hall, RM 300C, 370 Drillfield Drive, Blacksburg, VA 24061 USA; 2 Department of Mathematics, Virginia Tech, 434 McBryde Hall, 225 Stanger Street, Blacksburg, VA 24061 USA

**Keywords:** Fractions, Learning progression, Measurement concept

## Abstract

**Background:**

Fractions continue to pose a critical challenge for students and their teachers alike. Mathematics education research indicates that the challenge with fractions may stem from the limitations of part-whole concepts of fractions, which is the central focus of fractions curriculum and instruction in the USA. Students’ development of more sophisticated concepts of fractions, beyond the part-whole concept, lays the groundwork for the later study of important mathematical topics, such as algebra, ratios, and proportions, which are foundational understandings for most STEM-related fields. In particular, the *Common Core State Standards for Mathematics* call for students to develop measurement concepts of fractions. In order to support such concepts, it is important to understand the underlying mental actions that undergird them so that teachers can design appropriate instructional opportunities. In this study, we propose a learning progression for the measurement concept of fractions—one that focuses on students’ mental actions and informs instructional design.

**Results:**

A hierarchy of fraction schemes is charted outlining a progression from part-whole concepts to measurement concepts of fractions: (a) part-whole scheme (PWS), (b) measurement scheme for unit fractions (MSUF), (c) measurement scheme for proper fractions (MSPF), and (d) generalized measurement scheme for fractions (GMSF). These schemes describe concepts with explicit attention to the mental actions that undergird them. A synthesis of previous studies provides empirical evidence to support this learning progression.

**Conclusions:**

Evidence from the synthesis of a series of research studies suggests that children’s measurement concept of fractions develops through several distinct developmental stages characterized by the construction of distinct schemes. The mental actions associated with these schemes provide a guide for teachers to design instructional opportunities for children to advance their construction of a measurement concept of fractions. Specifically, the collection of quantitative studies suggest that students need opportunities to engage in activities that support two kinds of coordinations—the coordination of partitioning and iterating, and the coordination of three levels of units inherent in fractions. Instructional implications are discussed with example tasks and activities designed to provoke these coordinations.

## Background

Fractions continue to pose a critical challenge for American students and their teachers alike (Lamon 2007; National Mathematics Advisory Panel 2008; *Common Core State Standards for Mathematics [CCSSM]* 2010). Mathematics education research indicates that a central aspect of that challenge consists in addressing the limitations of part-whole concepts (Mack 2001; Pitkethly and Hunting 1996; Steffe and Olive 2010; Streefland 1991), which remain the central focus of fractions curriculum and instruction in the USA (Li et al. 2009; Watanabe 2007). Students’ development of more sophisticated concepts of fractions, beyond the part-whole concept, lays the groundwork for the later study of important mathematical topics, such as algebra (e.g., Hackenberg and Lee 2015), which is foundational for all STEM-related fields.

In a part-whole conception, students interpret fractions as a comparison of two numbers: the number of equal parts in the whole and the number of parts taken out of the whole to make the fraction. To understand the limitations of such a concept, consider an improper fraction, like 7/5. Because it is impossible to take seven parts out of five parts, students (and many teachers) reflexively convert such fractions to mixed numbers (e.g., 1 2/5) and avoid working with improper fractions altogether (Thompson and Saldanha 2003; Tzur 1999).

*Common Core State Standards for Mathematics* call on teachers to support more sophisticated concepts of fractions. By the end of fourth grade, students should understand non-unit fractions—even improper fractions—as multiples of a unit fraction (CCSSM 2010). For example, they should understand 3/5 as a fraction that is three times as big as 1/5. This is a laudable and ambitious goal that will involve supporting students’ reconceptualization of fractions from part-whole concepts to measurement concepts (Norton and Boyce 2013).

The part-whole and measurement concepts are among five fractions subconstructs studied by the Rational Number Project (Behr et al. 1983; Kieren 1980). Whereas the part-whole subconstruct involves understanding a proper fraction, *m*/*n*, as *m* equal parts taken out of *n* equal parts, the measurement subconstruct involves understanding that fraction as being *m* measures of the unit fraction, 1/*n*. The Fractions Project (Steffe and Olive 2010) has built on results of the Rational Number Project by specifying the mental actions—and their coordinations—that undergird various fraction concepts/subconstructs. For example, part-whole concepts rely on mental actions of partitioning and disembedding, with which students can project *n* equal parts in a continuous whole (partitioning) and pull out *m* of those parts without losing track of their containment within the whole (disembedding), resulting in the fraction *m*/*n*. One important mental action missing in the part-whole concept is iterating, with which students can use a unit fraction to measure off a longer part (e.g., a non-unit fraction or the whole). Whereas partitioning involves the projection of *n* equal parts into a continuous whole, iterating “involves mentally repeating a given length or area to produce a connected whole that is *n* times as big as the given part” (Wilkins and Norton 2011, p. 390). This coordination of actions involving iteration is essential in the measurement subconstruct (Kieren 1980), that is, understanding a non-unit fraction or whole as made up of iterations of a unit fraction. Brief descriptions of the mental actions included in our discussion are presented in Table 1.

**Table 1:** Description of mental actions

Mental action	Description
Partitioning	Projection of a composite unit into a continuous whole to create equally sized parts within the whole^a^
Iterating	Repeating a unit of length or area to produce a connected whole^a^
Disembedding	Taking parts out of a whole as separate units while maintaining their relationship with the whole
Splitting	Simultaneous composition of partitioning and iterating, as inverse actions
Units coordination	Maintaining relationships between the various levels of units within a mathematical experience; involves coordinating simpler actions, such as partitioning, iterating, and disembedding

^a^A whole unit can be continuous or discrete, but the discussion in this article focuses on a continuous whole. Also note that splitting and units coordination, as coordinations of other mental actions, are more advanced and separated in the table

The decomposition of students’ fraction concepts into the coordination of underlying mental actions has its roots in Piaget’s (1972) genetic epistemology. In that epistemology, mathematics is a product of psychology—derived from mental actions that are characterized by their composability and reversibility. For example, mental actions of partitioning and iterating can be composed with one another and can be considered as inverses of one another: the mental action of partitioning a continuous whole into *n* parts can be composed with the mental action of iterating one of those parts *n* times, and the result is the original whole (Wilkins and Norton 2011). Coordinations of mental actions like these form group-like structures that complement the sequential structures of schemes.

In this report, we share a hierarchy of fraction schemes that chart a progression from part-whole concepts to measurement concepts of fractions. These schemes describe concepts with explicit attention to the mental actions that undergird them. We then report on a synthesis of a series of previously conducted quantitative studies with regard to the hierarchy of schemes and related coordinations of actions. These studies provide empirical evidence for the learning progression for the measurement concept of fractions. Results should inform instruction while explaining the state of affairs in students’ development of fractions knowledge. Specifically, the collection of quantitative studies suggests that students need opportunities to engage in activities that support two kinds of coordinations—the coordination of partitioning and iterating, and the coordination of three levels of units inherent in fractions (the whole, the fraction, and its associated unit fraction).

## Theoretical framework

In addition to part-whole and measurement subconstructs, Kieren (1980) identified ratio, quotient, and operator as subconstructs in children’s constructions of rational number. We summarize the five subconstructs in Table 2. He described the part-whole subconstruct as a special case of the more general ratio subconstruct. Both subconstructs involve comparing numbers of equal parts, but the part-whole subconstruct has an additional distinction in specifying a particular whole. Whereas 3/5 and 30/50 constitute the same ratio, they might refer to different wholes (e.g., three heads in five coin flips or 30 heads in 50 coin flips); in a part-whole comparison, they would refer to the same whole partitioned into different numbers of equal parts. The quotient subconstruct again refers to two numbers, but in this case, fractions are understood as the result of dividing one whole number by the other whole number.

**Table 2:** Five subconstructs of rational number

Part-whole	Understanding a rational number or fraction, *m*/*n*, as *m* equal parts taken out of *n* equal parts
Quotient	Understanding a rational number or fraction, *m*/*n*, as dividing a quantity *m* into *n* equal parts
Measurement	Understanding a rational number or fraction, *m*/*n*, as being *m* measures of the unit fraction, 1/*n,* or *m* iterations of 1/*n*
Ratio	Understanding a rational number or fraction, *m*/*n*, as a relationship between the two quantities *m* and *n*, where either *m* + *n* = *whole* (part-part relationship) or *n* = *whole* (part-whole relationship, see part-whole subconstruct)
Operator	Understanding a rational number or fraction, *m*/*n*, as a function that multiplicatively maps a given quantity to another quantity, i.e., it operates on the given quantity

Based on Kieren (1980)

The measurement subconstruct involves determining the fractional size of one magnitude (e.g., length or area) relative to another by determining the number of times one fits into the other. In a longitudinal study of the five subconstructs, Lamon (2007) found that instruction based on the measurement subconstruct supported the most robust conceptions of fractions, in terms of connecting to other subconstructs. Specifically, she found that, for many students, the operator subconstruct naturally arose from the measurement subconstruct. The operator is a particularly advanced subconstruct in which fractions refer to a multiplicative mapping, such as mapping a figure to a figure that is 2/3 of the original size.

In the remainder of this section, we describe a progression of fraction schemes and related mental actions that elucidate students’ constructions of robust fraction concepts. Those schemes centrally rely upon coordinated mental actions, including those involved in coordinating multiple levels of units.

## Fraction schemes

**Fig. 1:**
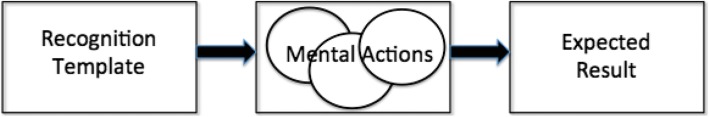
Three-part structure of a scheme

Based on Piaget’s (1972) genetic epistemology, von Glasersfeld (1995) described schemes as three-part structures through which students make sense of their experiences (see Fig. 1): a recognition template that triggers a sequence of mental actions, the mental actions themselves, and an expected result from carrying out those actions. A mathematical experience consists of a situation in which particular kinds of mental actions are called upon—reversible and composable mental actions, such as partitioning and iterating. Here, we describe a progression of four fraction schemes that involve such mental actions: the part-whole scheme (PWS), the measurement scheme for unit fractions (MSUF), the measurement scheme for proper fractions (MSPF), and the generalized measurement scheme for fractions (GMSF). Brief descriptions of these schemes are presented in Table 3.

**Table 3:** Progression of four fraction schemes

Scheme	Associated actions
Part-whole scheme (PWS)	Produce any proper fraction, *m*/*n*, from a given whole by partitioning the whole into *n* parts and disembedding *m* of the parts.
Measurement scheme for unit fractions (MSUF)	Determine the fractional size of a unit fraction, relative to a given unpartitioned whole, through iterating the unit fraction to produce the whole.
Measurement scheme for proper fractions (MSPF)	Produce an unknown whole from a proper fraction, *m*/*n*, by partitioning the proper fraction of the whole into *m* equal parts to produce a unit fraction, 1/*n*, and iterating 1/*n* to determine the whole.
Generalized measurement scheme for fractions (GMSF)	Produce an unknown whole from *any* fraction, *m*/*n*, *including improper fractions*, by partitioning the fraction into *m* equal parts to produce a unit fraction, 1/*n*, and iterating 1/*n* to determine the whole.

### Part-whole scheme

**Fig. 2:**
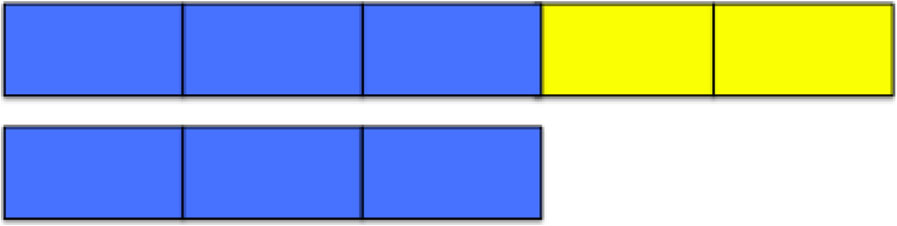
Disembedding three-fifths from the whole

In addition to partitioning, the PWS utilizes the mental action of disembedding parts from a whole. Students use partitioning to break a continuous whole into a specified number of equal parts; they use disembedding to take any number of those parts as both a separate collection and, at the same time, as parts within the whole. When students do not disembed, they sometimes consider the fraction to be the entire picture, rather than the sub-collection, or in considering the sub-collection, they might lose track of the whole and take the sub-collection as its own whole (Olive and Vomvoridi 2006). For example, they might name the entire picture of three parts shaded within five parts as the whole, or they might name the three parts pulled out of the five parts as three-thirds (see Fig. 2). On the other hand, students who have constructed a PWS can make any proper fraction, *m*/*n*, from a given whole by partitioning the whole into *n* parts and disembedding *m* of them. Furthermore, on the basis of disembedding, they can appropriately identify the fraction as the *m* parts and appropriately name that fraction as *m*/*n*.

### Measurement scheme for unit fractions

**Fig. 3:**
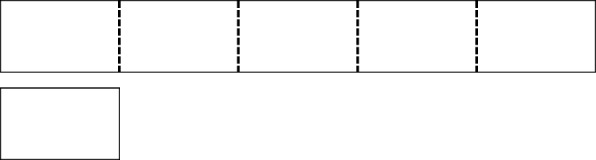
Determining the unit fractional size of a part relative to the whole

The clearest distinction between measurement schemes and the PWS lies in their use of the mental action of iteration. In the simplest case—the case of unit fractions—students can iterate a smaller magnitude within a larger magnitude to determine the number of times the former fits into the latter (Steffe and Olive 2010; Steffe 2002). They can also appropriately name the unit fractional size of the smaller magnitude relative to the larger magnitude, based on the number of iterations. Specifically, they understand the reciprocal relationship between the number of iterations and the unit fractional size. For example, consider Fig. 3.

Determining the fractional size of the small bar relative to the long (unpartitioned) bar is a situation that would fit in the recognition template of the MSUF. For students who have constructed such a scheme, the situation would trigger the mental action of iterating the small bar within the long bar five times. The activity of iterating suggests a partitioning of the long bar into five parts (represented by the dotted lines), and the small bar is understood as one part disembedded from those five parts.

### Measurement scheme for proper fractions

Students who have constructed a MSUF can generalize that way of operating to all proper fractions. However, with non-unit fractions (e.g., 3/5), they need to iterate the unit fraction, as if it were a unit of 1, to measure both the whole and the non-unit fraction. For example, in producing the fraction, 3/5, as a measure, students must understand the 1 to 5 relationship between the unit fraction (1/5) and the whole, just as they would with the MSUF. Additionally, they would need to produce 3/5 from 1/5 by iterating the 1/5 part three times, producing 3/5 as a composite unit—a unit composed of three iterations of the 1/5 part.

**Fig. 4:**
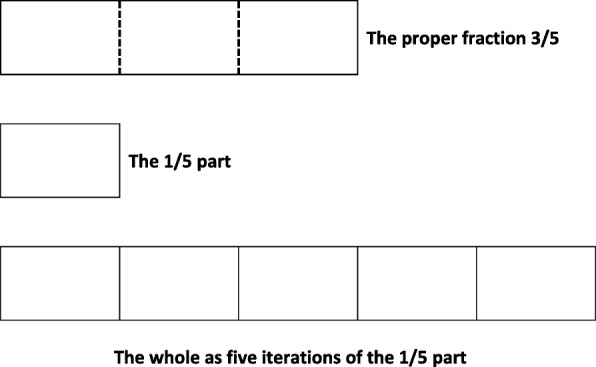
Producing the whole from a proper fraction

Just as the iterative relationship between the unit fraction and the whole suggests an inverse relationship of partitioning the whole into unit fractions (as described with regard to the MSUF), the iterative relationship between the unit fraction (1/5) and the non-unit proper fraction (3/5) suggests an inverse relationship of partitioning that composite unit (3/5) in three (1/5) units. Thus, students operating with a MSPF can reproduce the whole from the proper fraction by partitioning the proper fraction into three equal parts (represented by the dotted lines in Fig. 4) and iterating one of those (1/5) parts five times (see Fig. 4). Note, however, that in treating the unit fraction as a unit of 1, students operating with a MSPF are not maintaining the relationships of all three units (1/5, 3/5, and the whole) at once. Rather, they assimilate the goal of making the whole from a 3/5 part as a goal of making 5 from 3.

### Generalized measurement scheme for fractions

**Fig. 5:**
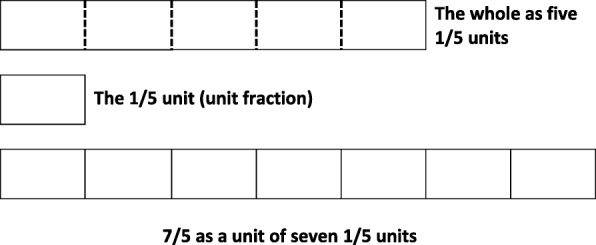
7/5 as a unit of seven units of 1/5, five of which comprise the whole

With the MSPF, there are two two-level relationships to maintain in the fraction *m*/*n*: the 1-to-*n* relationship between the unit fraction and the whole and the 1-to-*m* relationship between the unit fraction and the (non-unit) proper fraction. Students can work across these two two-level relationships, sequentially, by referencing the whole. However, when the fraction exceeds the whole—as in the case of improper fractions—the whole is easily lost. Thus, to reliably work with improper fractions, students must maintain all three levels of units at once. That is not to say that the GMFS only applies to improper fractions. Students with a GMSF begin to understand *all* fractions as “numbers in their own right” (Hackenberg 2007, p. 27), wherein *m*/*n* represents relationships between all three levels of units, simultaneously. Still, the distinction is clearest when students are working with improper fractions, like 7/5 (see Fig. 5).

Students might be able to produce improper fractions from a given whole before they have constructed a GMSF. However, these students will often refer to the fraction they produced as 7/7 or even 5/7, because they cannot maintain all three levels of units at the same time. In the next section, we describe the coordinations of mental actions involved in maintaining various levels of units. We also describe how partitioning and iterating are coordinated with one another within a new mental action called splitting.

## Splitting and units coordination

In addition to the three-part structure of schemes, Piaget identified another kind of cognitive structure—one that is particular to mathematics. These group-like structures describe how mental actions are related to one another, independent of the situations in which they might be applied. Here, “group” refers to a simple mathematical structure wherein mental actions can be composed with one another and every mental action has an associated inverse action that undoes it (Piaget 1970). Consider, for example, the mental actions of partitioning and iterating (a brief description of the mental actions discussed here is provided in Table 1).

### Splitting

As noted previously, partitioning and iterating comprise mental actions that can be composed with one another and, more specifically, form inverses of one another so that, when composed, they undo one another. Thus, they form a simple group-like structure, called splitting (Steffe 2002; Wilkins and Norton 2011). The organization of partitioning and iterating as inverse elements within this structure is what enables students to reverse their reasoning when working with the MSPF (producing the whole from a given proper fraction as previously described; Hackenberg 2010).

Note that splitting involves more than sequentially applying iterating and partitioning actions. Because partitioning and iterating are organized within a single structure, students who split can anticipate the result of partitioning an iteration or iterating a partition (Wilkins and Norton 2011). Returning to the example of 3/5, students with a MSPF understand it as three iterations of a 1/5 part and, because they can split, anticipate that partitioning a 3/5-bar into three of those parts.

### Units coordination

As with splitting, units coordination structures refer to organizations of mental actions within structures for composing and reversing them. Several researchers have alluded to units coordination structures as serving important roles in students’ numerical cognition (e.g., Behr et al. 1983; Lamon 2007), and they play a particularly important role in fraction schemes, as described by Steffe and Olive (2010). Here, we elucidate one kind of units coordination structure involved in conceptualizing fractions—one for coordinating three levels of fractional units.

Understanding non-unit fractions as numbers involves maintaining relationships between three levels of units: the fraction itself, as a quantity; the unit fraction used to measure it; and the referent whole. Maintaining all three levels of units when working with fractions as numbers requires a structure for organizing their relationships—the *n*-to-1 relationship between the unit fraction (*1*/*n*) and the whole, and the *m*-to-1 relationship between the non-unit fraction (*m*/*n*) and the unit fraction. For this reason, a units coordinating structure for simultaneously coordinating three levels of fractional units is prerequisite for the GMSF.

## Purpose

The purpose of this study is to highlight the learning progression outlined above based on the hierarchy of fraction schemes (Steffe and Olive 2010). We draw on a series of studies (Norton and Wilkins 2009, 2010, 2012, 2013; Norton et al. 2018; Wilkins and Norton 2011) that have hypothesized and tested relationships among smaller sets of schemes and mental actions associated with the fractions hierarchy (Steffe and Olive 2010). We synthesize the evidence from these studies to provide support for the learning progression for the measurement concept of fractions.

In order to focus on the measurement concept, we have renamed some of the schemes and combined others to remain consistent with the proposed theoretical framework. For reference to the previous research, the measurement scheme for unit fractions (MSUF) aligns with the partitive unit fraction scheme (PUFS; Steffe and Olive 2010). The measurement scheme for proper fractions (MSPF) represents a combination of the partitive fraction scheme (PFS) and the reversible partitive fraction scheme (RPFS) (Steffe and Olive 2010; also see Norton and Wilkins 2010 for an examination of the partitive fraction schemes). In previous research, these two schemes (PFS and RPFS) were investigated separately to remain consistent with the hierarchy proposed by Steffe and Olive (2010). This combination resulting in the MSPF represents a theoretical criterion for the interiorization of a scheme—that the mental actions of the scheme are both composable and reversible (Piaget 1970). Finally, the generalized measurement scheme for fractions (GMSF) aligns with the iterative fraction scheme (IFS; Steffe and Olive 2010). The naming of the part-whole scheme and the various mental actions remain consistent with Steffe and Olive (2010).

## Model of the learning progression for the measurement concept of fractions

**Fig. 6:**
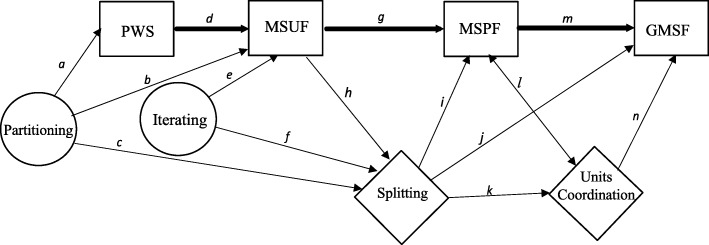
Hierarchy of schemes and mental actions

In Fig. 6, we present a model of the learning progression for developing measurement concepts of fractions. The four schemes are represented with rectangles and are presented horizontally. The thicker single-headed arrows represent the order of the developmental progression of the schemes. The thinner single-headed arrows represent a developmental prerequisite. For example, the partitioning and iterating actions are presented in circles with thinner single-headed arrows representing their basis as developmental prerequisites for the construction of subsequent fraction schemes and the more advanced mental action of splitting, presented in a diamond.

Splitting is shown to be a developmental prerequisite for the construction of the MSPF and three levels of units coordination (also in a diamond). Splitting is represented as a necessary prerequisite for the construction of the MSPF and the GMSF, with the coordination of three levels of units also necessary for the GMSF. The MSUF is represented as a developmental prerequisite of splitting.

The construction of the MSPF is associated with the interiorization of three levels of units coordination, but they develop alongside one another (represented by a two-headed arrow), that is, there is a relationship between the construction of a MSPF and units coordination, but the order of development is not directional. The MSPF does involve three levels of units, but because the units are within the whole, it is possible for children to coordinate these units two at a time. As previously discussed, the simultaneous coordination of three levels of units is necessary for the construction of the GMSF (represented by a thin single-headed arrow).

## Methods and background of studies

Together, the studies that we draw on included more than 300 students in grades 5, 6, 7, and 8. Students were from schools in the midwestern (Norton and Wilkins 2009) and southeastern (Norton and Wilkins 2010, 2012, 2013; Wilkins and Norton 2011) USA, as well as from China (Norton et al. 2018). Research procedures in these studies were approved by the Institutional Review Board for research involving human subjects. Tasks used in these studies were created to elicit the different ways of operating associated with the different fraction schemes and associated mental actions (see, e.g., Norton and Wilkins 2012; Norton et al. 2018; Wilkins and Norton 2011; Wilkins et al. 2013). In these studies, sets of four items designed specifically for each scheme or operation were used to determine whether students had constructed each scheme and operation. Relationships between schemes and operations were analyzed using descriptive statistics and measures of association (e.g., gamma statistic). For more specific information on the design and analyses used in these studies, see Wilkins and Norton (2011).

## Results and discussion

In Table 4, we summarize the percentage of students within each of the studies who had indications of having constructed each of the particular schemes and mental actions. We use these percentages as initial evidence of the developmental progression of schemes outlined above. However, these percentages alone do not substantiate the interrelationships shown in the model in Fig. 6. Further evidence will be discussed, drawing on additional findings from within the separate studies. These additional findings from within the separate studies are documented based on statistical analyses that consider and test for the interrelationships among the different schemes and mental actions.

**Table 4:** Percentage of students with indication of fraction schemes and associated mental actions by grade

	Grade 5^a^ (*N* = 44)	Grade 5^b^ (*N* = 45)	Grade 6^a^ (*N* = 40)	Grade 6^b^ (*N* = 31)	Grade 6^c^ (*N* = 66)	Grade 6^d^ (*N* = 49)	Grade 7^e^ (*N* = 56)	Grade 7^g^ (*N* = 49)	Grade 8^h^ (*N* = 58)
Scheme/action	%	%	%	%	%	%	%	%	%
Partitioning					88				
Part-whole	58	80	78	100					
Iterating					86				
MSUF	52	67	70	81	61	59	61	65	
Splitting	34	42	36	77	44	41	55	63	66
MSPF	14	16	18	19	(17)		13^f^		19
Units coordination									26
GMSF	14		20						12

Percentage in parentheses represents only the composability component of the MSPF and does not include tasks testing for reversibility

^a^From Norton and Wilkins 2009

^b^From Norton et al. 2018

^c^From Wilkins and Norton 2011

^d^From Norton and Wilkins 2013, subset of children in Wilkins and Norton 2011

^e^From Norton and Wilkins 2010

^f^Not previously published, but associated with students in Norton and Wilkins 2010

^g^From Norton and Wilkins 2013

^h^From Norton and Wilkins 2012

Looking across the four fraction schemes PWS ➔ MSUF ➔ MSPF ➔ GMSF, the percentages provide evidence for the hierarchical development of the four schemes (see Fig. 6 and Table 4). For example, consider column one for a sample of fifth graders (Norton and Wilkins 2009). The percentage decreases from 58% for the PWS to 52% for the MSUF and 14% for the MSPF and then 14% for the GMSF. Looking across different studies, the evidence is most pronounced for the first three schemes (PWS ➔ MSUF ➔ MSPF); in all cases, the percentage of students within each study having constructed the scheme decreases as the scheme becomes more advanced. Overall, these percentages provide evidence in support of the learning progression for the development of the measurement concept of fractions.

Further considering the developmental progression from PWS to MSUF, this relationship was hypothesized and tested in a study of 76 fifth and sixth graders in China (see Fig. 6, path *d*; see Table 4, columns 2 and 4; Norton et al. 2018). As hypothesized, the construction of a PWS was documented to developmentally precede the construction of the MSUF.

The interrelationships among partitioning, iterating, splitting, and the MSUF were hypothesized and tested in a study of 66 sixth graders (Wilkins and Norton 2011; see Fig. 6 paths *b*, *c*, *e*, *f*, and *h*; see Table 4, column 5). As hypothesized, partitioning and iterating were both found to developmentally precede MSUF and splitting. Furthermore, the construction of a MSUF, as a result of composing iterating and partitioning, was documented to mediate the construction of splitting, which represents an important developmental transition. The importance of this transition was further highlighted in another study (Norton and Wilkins 2013) in which it was documented that students who had constructed an MSUF by the end of sixth grade were approximately 13 times more likely to construct splitting by the end of seventh grade than the students who had not constructed an MSUF. These findings emphasize the importance of developing even the earliest forms of the measurement concept.

The construction of splitting and the coordination of units are important for their roles in the construction of more advanced fraction schemes and mental actions. The interrelationships among splitting, units coordination, the MSPF, and the GMSF are shown in Fig. 6 and were hypothesized and tested in a study of 58 eighth graders (Norton and Wilkins 2012; see Fig. 6, paths *i*, *j*, *k*, *l*, *m*, and *n*; see Table 4, column 9). As hypothesized, splitting was documented as a developmental prerequisite for the construction of a MSPF (also see Table 4 with reference to Norton and Wilkins 2009 and Norton et al. 2018) and a GMSF. Findings from this study also provided evidence that splitting precedes the simultaneous coordination of three levels of units. In addition, as hypothesized, the construction of a MSPF was shown to precede the construction of a GMSF (also see Norton and Wilkins 2009, 2012).

Key to the distinction between the construction of a MSPF and the GMSF, children’s simultaneous coordination of units was documented as a developmental prerequisite for the construction of a GMSF; however, simultaneous coordination of units was not found to be a developmental prerequisite for the construction of a MSPF. Although the construction of a MSPF was found to be positively related with the simultaneous coordination of three levels of units, several students were documented to have constructed a MSPF without simultaneously coordinating three levels of units. However, no students were found to have constructed a GMSF without first being able to simultaneously coordinate three levels of units. This finding is consistent with the proposed learning progression, in that some children who are only able to coordinate a three-level structure sequentially as two two-level structures can still solve tasks like that presented in Fig. 4 (a task designed to indicate the construction of a MSPF). However, this sequential coordination of units is not sufficient for the construction of a GMSF, which is required for tasks like the one presented in Fig. 5.

In summary, splitting affords students the ability to reverse their reasoning with fractions as measures, which enables them to construct the MSPF. Students who are able to simultaneously coordinate three levels of units are then poised to reorganize their MSPF to deal with fractions of any size, including improper fractions, that is, to construct a GMSF. At this point, students are able to produce any fraction from a given whole or recreate the whole from a fraction of any size.

Finally, the developmental hierarchy between MSUF and MSPF was hypothesized and tested in several studies across grades 5, 6, and 7 (Norton and Wilkins 2009, 2010; Norton et al. 2018; see Fig. 6, path *g*; Table 4, columns 1, 2, 3, 4, and 7). As discussed previously, in all cases, the percentage of students having constructed a MSUF was much larger than the percentage of students having constructed a MSPF. In addition, in each of these studies, it was statistically documented that the MSUF preceded the development of the MSPF. Therefore, it is clear from these studies that there are developmental differences for students when dealing with measurement situations associated with proper fractions as compared to unit fractions. This is interesting from an instructional perspective as the same has not been found to be true for students working in part-whole situations, that is, there are no operational differences between unit and proper fractions when dealing with part-whole situations (Norton and Wilkins 2009).

## Conclusions

Kieren’s (1980) five subconstructs (see Table 2) provide a way of understanding the many facets of rational number and point to the importance of “developing mechanisms for building rational number concepts” (p. 127) that can inform instruction. We have provided evidence for underlying mechanisms, in the form of schemes and mental actions, particularly with regard to the measurement subconstruct. Kieren (1980) alluded to the importance of partitioning and iterating in the formation of the measurement construct, and we highlight their importance in constructing splitting and the coordination of three levels of units that make possible the construction of the more advanced measurement schemes (Norton and Wilkins 2012; Wilkins and Norton 2011; Hackenberg 2007). We highlight these mechanisms as they distinguish children’s mental actions with unit fractions, proper fractions, and improper fractions.

As Lamon (2007) documented in her study, instruction on the measurement subconstruct leads to the most robust conceptions of fractions. We have outlined and provided support for a learning progression from the part-whole subconstruct to the construction of a generalized measurement concept of fractions, the GMSF. Kieren (1980) highlighted the interaction among the subconstructs, and with the construction of a GMSF, children are further poised to understand the operator subconstruct (Lamon 2007; Norton and Wilkins 2012; cf. Thompson and Saldanha 2003)—an understanding that affords children the ability to map one rational number to another in a multiplicative way (Hackenberg and Tillema 2009).

The learning progression outlined above provides a theoretical guide to design instructional tasks that provide opportunities for students to construct the schemes and mental actions necessary to build a measurement concept of fractions. We offer sample tasks and activities that could fill out a trajectory with learning opportunities for students to move through the progression. We start with example tasks meant to move students from a part-whole conception of fractions to construct a MSUF. This can be done by engaging students in tasks involving the production of connected multiples from a given unit (e.g., Steffe 2002). Steffe concluded that such tasks create a bridge between students’ use of iterating in discrete, whole-number contexts (e.g., part-whole) to their use of iteration in continuous, fractional contexts (e.g., MSUF).

**Fig. 7:**
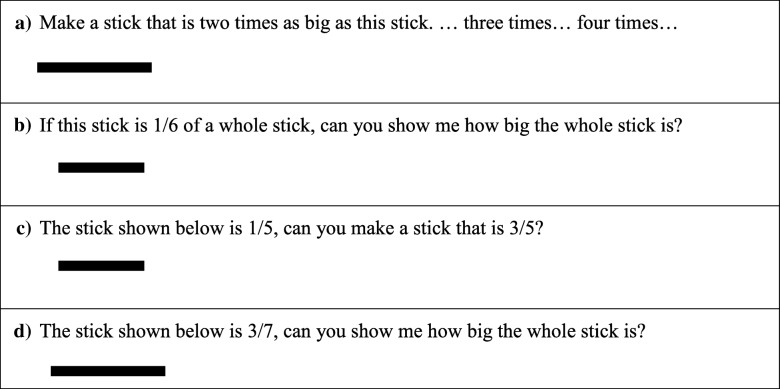
**a**–**d** Examples of tasks for transitioning children from a PWS to MSUF to MSPF

Figure 7 illustrates such tasks. In Fig. 7a, children are asked to create multiples of the stick through iteration. There is no fractional language in the task, but solving the task involves measuring one length with a given unit of length—a precursor to measuring the whole or a non-unit fraction, with a unit fraction, as a unit of measure. For the task shown in Fig. 7b, fraction language connects students’ iterating activity to a specific magnitude (1/6) to determine the number of times the unit fraction fits into the whole—essential understanding for the construction of a MSUF.

To help transition children from a MSUF to a MSPF, a task like that in Fig. 7c would require children to understand the 1 to 5 relationship between the unit fraction (1/5) and the whole, as with the MSUF, but additionally, they would need to create 3/5 from 1/5 by iterating the 1/5 unit three times, producing 3/5 as a composite unit—a unit composed of three 1/5 parts. A task like that shown in Fig. 7d would challenge students to reverse that reasoning to create the unit fraction (1/7) associated with 3/7 and iterate it to create the whole (Hackenberg 2010). This level of thinking and activity characterizes the understandings associated with a MSPF.

In addition to tasks like those in Fig. 7, recent development of educational video games offer dynamic environments in which to help children coordinate their actions of partitioning and iterating (Aslan et al. 2012; Norton et al. 2014). In the game CandyFactory (version 2.0; Aslan et al. 2012), children work for a company filling orders for candy that meet the specific requirements, through the use of partitioning and iterating. As highlighted earlier, splitting is necessary for students to reverse their partitive thinking and construct a MSPF. The construction of splitting seems to develop through continued opportunities for children to coordinate their partitive thinking with unit fractions (Norton and Wilkins 2013; Steffe 2002). As discussed earlier, the MSUF involves the sequential use of partitioning and iterating. Through continued opportunities to partition figures to create units that can then be iterated to reform the whole and at the same time iterate units to create a whole that can then be partitioned to reform a unit, students begin to see these sequential mental actions as a single simultaneous mental action, splitting (see Table 1). Tasks like those in Fig. 7 and CandyFactory not only support the development of a MSUF but also lay the groundwork for the construction of splitting.

Finally, in order to construct a GMSF, children must first be able to coordinate at least three levels of units at a time. Giving children opportunities to solve tasks that involve multiple levels of units can lead to growth in children’s coordination of units (Norton and Boyce 2015). Norton and Boyce (2015, p. 216) systematically designed scenarios involving four levels: boxes of cups, number of chips in a cup, and a price for a chip, e.g., 2 chips in a cup, 5 cups in a box, and 2 cents per chip. They then asked questions that involved the manipulation of the multiple units: “How much is a box worth?”; “If you have 14 cents, how many more chips do you need to make a box?” (p. 216). Similar tasks have also been developed as part of an educational video game, CandyDepot (version 2.0), in which children work for a shipping company packing bars in bundles and bundles in boxes in the most efficient way (Aslan et al. 2013; also see Norton et al. 2015).

Through the careful use of tasks and activities that focus on the underlying mechanisms or mental actions that support the development of fraction concepts, children can construct more robust concepts of fractions. In particular, by purposefully choosing tasks that provoke children’s actions associated with the coordination of partitioning and iterating, as well as the coordination of multiple levels of units, children can construct powerful measurement concepts for fractions. Moreover, the power of these measurement concepts extends beyond the domain of fractions.

CCSSM (2010) calls for children to extend their understanding of the number line to include rational numbers. In that context, unit fractions become units of measure, just as described here for the MSPF and the GMSF. Whereas measurements in units of 1 generate the number line for natural numbers, measurements in units of 1/*n* extend that line to all positive rational numbers (Kallai and Tzelgov 2009). Additionally, the MSPF and GMSF open doors for students to develop algebraic reasoning, wherein they operate on unknown quantities (Booth and Newton 2012; Hackenberg and Lee 2015). Like 1 and unit fractions, an unknown quantity, *x*, becomes a unit that students can partition and iterate, while coordinating associated units, to build relationships that they can represent with algebraic equations.

## Abbreviations

GMSF: Generalized measurement scheme for fractions
IFS: Iterative fraction scheme
MSPF: Measurement scheme for proper fractions
MSUF: Measurement scheme for unit fractions
PFS: Partitive fraction scheme
PUFS: Partitive unit fraction scheme
PWS: Part-whole scheme
RPFS: Reversible partitive fraction scheme
